# Interleukin-8 Secreted by Glioblastoma Cells Induces Microvascular Hyperpermeability Through NO Signaling Involving S-Nitrosylation of VE-Cadherin and p120 in Endothelial Cells

**DOI:** 10.3389/fphys.2019.00988

**Published:** 2019-08-08

**Authors:** Anita Guequén, Patricia Zamorano, Francisco Córdova, Tania Koning, Angelo Torres, Pamela Ehrenfeld, Mauricio P. Boric, Flavio Salazar-Onfray, Julie Gavard, Walter N. Durán, Claudia Quezada, José Sarmiento, Fabiola A. Sánchez

**Affiliations:** ^1^Instituto de Inmunología, Facultad de Medicina, Universidad Austral de Chile, Valdivia, Chile; ^2^Instituto de Bioquímica y Microbiología, Facultad de Ciencias, Universidad Austral de Chile, Valdivia, Chile; ^3^Instituto de Histología, Anatomía y Patología, Facultad de Medicina, Universidad Austral de Chile, Valdivia, Chile; ^4^Centro Interdisciplinario de Estudios del Sistema Nervioso (CISNe), Universidad Austral de Chile, Valdivia, Chile; ^5^Departamento de Fisiología, Pontificia Universidad Católica de Chile, Santiago, Chile; ^6^Instituto Milenio de Inmunología e Inmunoterapia, Facultad de Medicina, Universidad de Chile, Santiago, Chile; ^7^Team SOAP, Signaling in Oncogenesis, Angiogenesis and Permeability, INSERM, CNRS, Institut de Cancérologie de l’Ouest, Université de Nantes, Nantes, France; ^8^Department of Pharmacology, Physiology and Neuroscience, Rutgers New Jersey Medical School, The State University of New Jersey, Newark, NJ, United States; ^9^Instituto de Fisiología, Facultad de Medicina, Universidad Austral de Chile, Valdivia, Chile

**Keywords:** adherens junction, glioblastoma, endothelial permeability, S-nitrosylation, VE-cadherin

## Abstract

Glioblastoma is a highly aggressive brain tumor, characterized by the formation of dysfunctional blood vessels and a permeable endothelial barrier. S-nitrosylation, a post-translational modification, has been identified as a regulator of endothelial function. In this work we explored whether S-nitrosylation induced by glioblastoma tumors regulates the endothelial function. As proof of concept, we observed that S-nitrosylation is present in the tumoral microenvironment of glioblastoma in two different animal models. Subsequently, we measured S nitrosylation and microvascular permeability in EAhy296 endothelial cells and in cremaster muscle. *In vitro*, conditioned medium from the human glioblastoma cell line U87 activates endothelial nitric oxide synthase, causes VE-cadherin- S-nitrosylation and induces hyperpermeability. Blocking Interleukin-8 (IL-8) in the conditioned medium inhibited S-nitrosylation of VE-cadherin and hyperpermeability. Recombinant IL-8 increased endothelial permeability by activating eNOS, S-nitrosylation of VE-cadherin and p120, internalization of VE-cadherin and disassembly of adherens junctions. *In vivo*, IL-8 induced S-nitrosylation of VE-cadherin and p120 and conditioned medium from U87 cells caused hyperpermeability in the mouse cremaster muscle. We conclude that eNOS signaling induced by glioma cells-secreted IL-8 regulates endothelial barrier function in the context of glioblastoma involving S-nitrosylation of VE-cadherin and p120. Our results suggest that inhibiting S-nitrosylation may be an effective way to control and/or block damage to the endothelial barrier and prevent cancer progression.

## Introduction

Glioblastoma (GBM) is a highly aggressive brain tumor that currently lacks effective treatment (Rahman et al., 2010; Lim et al., 2011; Torres et al., 2017). The malignancy degree in GBM correlates with enhanced expression and activity of the three isoforms of nitric oxide synthase in tumoral cells and in endothelium (Cobbs et al., 1995; Bakshi et al., 1998). S-nitrosylation is a post-translational modification induced by nitric oxide (NO) in free-thiol cysteine in proteins (Stamler et al., 1992). In GBM, caspase-3 and PTEN in tumoral cells are the only two identified targets of S-nitrosylation that contribute to glioma expansion (Yu et al., 2005; Shen et al., 2016), however, the contribution of S-nitrosylation of endothelial proteins to GBM physiopathology has not been investigated yet. A major feature of GBM resides in its leaky endothelial barrier that contributes to angiogenesis and edema (Dubois et al., 2014). In this regard, brain endothelial cells cultured with conditioned medium from GBM cells show increased permeability, a characteristic associated with increased levels of interleukin-8 (IL-8) (Dwyer et al., 2012). The integrity of the endothelial barrier depends largely on adherens junctions, which consist of vascular endothelial-cadherin (VE-cadherin) forming a complex with p120, β-catenin, α-catenin and γ-catenin (Weis and Nelson, 2006; Giannotta et al., 2013; Radeva and Waschke, 2017). Our group and others have reported that pro-inflammatory cytokines stimulated S-nitrosylation of adherens junction proteins leading to internalization of these proteins resulting in destabilization of the endothelial barrier (Thibeault et al., 2011, Marin et al., 2012; Guequen et al., 2016). However, whether or not this post-translational modification participates in the endothelial permeability alterations associated to GBM remains unknown. The aim of this work was to investigate whether S-nitrosylation participates in vascular hyperpermeability induced by conditioned medium from GBM cell line U87 (U87-CM) and IL-8. Our work shows that IL-8, present in U87-CM, activates the endothelial nitric oxide synthase -S-nitrosylation pathway, leading to S-nitrosylation of VE-cadherin and endothelial hyperpermeability. *In vivo*, in the mouse cremaster model, IL-8 induced S-nitrosylation of VE-cadherin and p120 and U87-CM increased endothelial permeability through eNOS signaling. Our data support the concept that S-nitrosylation is a key regulator of endothelial barrier in GBM microenvironment.

## Materials and Methods

### Reagents

We obtained recombinant IL-8 using previously established protocols (Joseph et al., 2010) and administered it at 100 nM. 1H-[1,2,4]oxadiazolo[4,3-a]quinoxalin-1-one (ODQ), NG-methyl-L-arginine (L-NMA) and fluorescein isothiocyanate-labeled dextran 70 (FITC-Dx-70; molecular weight: 70,000 Da), were from Sigma-Aldrich (St. Louis, MO, United States). L-NMA (300 μM) and ODQ (10 μM), were added 1 h and 10 min, respectively, before agonist application.

### Antibodies

Mouse anti-eNOS, mouse anti-eNOS-Ser1177 and mouse anti-p120 were from BD Transduction Laboratories (San José, CA). Goat anti VE-cadherin was from Santa Cruz (Dallas, TX, United States). Mouse anti-IL-8 blocking antibody (MAB208) was from R&D Systems (Minneapolis, MN, United States). Mouse anti-β-actin, rabbit anti- β-catenin and rabbit anti-S-Nitroso-Cysteine (SNO-Cys), were from Sigma (St. Louis, MO, United States).

### Rat Brain Tumors

The generation of rat brain tumors (Barth and Kaur, 2009; Huszthy et al., 2012) was approved by the Institutional Animal Care and Use Committee at the Universidad Austral de Chile according to the NIH Guide for the Care and Use of Laboratory Animals. Sprague Dawley rats were maintained under standard laboratory conditions: 12 h light-dark cycle, with food and water *ad libitum*. For surgery, 8-week-old male rats (200–250 g) were anesthetized via intraperitoneal administration of a mixture of ketamine (100 mg/kg)/xylazine (10 mg/kg) and then placed in a stereotactic frame (Micro Control Instrument Ltd., United Kingdom). A burr hole was drilled in the skull 2 mm rostral and 1.5 mm right lateral to the bregma. 2 × 10^6^ C6 rat glioma cells were inoculated in 4 μL (5 nL/s) using a microliter Hamilton^®^ syringe (Hamilton, Reno, NV, United States) at 7 mm of depth. Drilled hole and skin were closed with dental cement and 3-O mononylon ethilon suture, respectively. At day fourteen post-inoculation, signs of neurological disorders (motor incoordination, loss of appetite, decreased alertness, and head pressing) were evident so rats were euthanized by intraperitoneal administration of sodium thiopental (120 mg/kg) and pneumothorax. Finally, brains were removed for histopathologic analysis.

### Mouse Subcutaneous Tumors

NOD/SCID-IL2Rγ^*null*^ mice (No. 005557; The Jackson Laboratory©, United States) of 25 g were anesthetized [ketamine (100 mg/kg)/xylazine (10 mg/kg) via intraperitoneal] and inoculated subcutaneously with 1 × 10^5^ U87MG-GSCs at the left flank. Mice were maintained under standard laboratory conditions of 12 h light-dark cycle, with food and water *ad libitum* according the Institutional Animal Care and Use committee at the Universidad Austral de Chile. 17 day’s post-inoculation, mice were euthanized with Sodium Thiopental (120 mg/kg via intravenous) and subcutaneous tumors were removed and fixed for histopathological analysis.

### Histopathologic Analysis

Tumor samples were fixed with Bouin, embedded in paraffin wax, cut into 5 μm sections and adhered to poly-L-lysine-coated slides. Tissue sections were dewaxed and rehydrated. Sections were treated with absolute methanol/3% H_2_O_2_ for 5 min to quench endogenous pseudoperoxidase activity, rinsed in distilled water and then in Tris–HCl buffer. After blocking non-specific binding for 15 min with horse serum (Vecton, Carpinteria, CA, United States), tissue sections were incubated with anti-S-Nitroso-Cysteine. The antibody was reconstituted and diluted in 0.01 M PBS containing 1% IgG-free bovine serum albumin (BSA; Sigma-Aldrich, St. Louis, MO, United States) and 0.2% NaN3. Bound antibodies were detected using the R.T.U VECTASTAIN Detection Kit (Vector Laboratories) and peroxidase was visualized with Liquid-3,3′diaminobenzidine (DAB) + Substrate Chromogen System (DAKO). Controls included omission of primary antibody. Slides were counterstained with hematoxylin-eosin and mounted with mounting medium (DAKO). Cells were visualized using a light microscope (Zeiss).

### Cell Culture and Preparation of Conditioned Media

Immortalized human endothelial cells, EAhy926 (derived from human umbilical vein; kindly donated by Dr. C. J. S. Edgell, University of North Carolina, Chapel Hill, NC, United States) (Edgell et al., 1983) were grown in basal media composed of: Dulbecco’s modified Eagle’s medium (DMEM) supplemented with 10% (v/v) fetal bovine serum, 2 mM L-glutamine, 100 U/ml penicillin, 100 μg/ml streptomycin and 2.5 μg/ml fungizone and supplemented with HAT (sodium hypoxanthine, aminopterin, and thymidine). Human GBM cell line U87 was maintained in DMEM supplemented with 10% FBS, 100 U/ml penicillin and 100 μg/ml streptomycin. For conditioned media, the three days’ confluent media from U87 cells was decanted and cleared by centrifugation at 1000 rpm for 5 min followed by filtration through 0.2 μm filter. U87-CM was then used immediately or stored at −80°C until use. To detect IL-8 in the conditioned medium ELISA was performed as described in Dwyer et al. (2012), according to the manufacturer’s instructions (Quantikine ELISA kit, R&D Systems, Biotechne, Lille, France).

### Western Blot Analysis

Confluent cells growing in a 60-mm plate were serum starved overnight. U87-CM or 100 nM IL-8 was applied to cells for different times. Cells were washed twice with ice cold PBS and scraped in 300 μL lysis buffer (50 mM Tris, 150 mM NaCl, 0.1 mM EDTA, 0.1 mM EGTA, 1% Triton X-100, and protease inhibitor mixture) and incubated on ice with shaking for 30 min. Lysates were obtained by centrifugation at 10,000×*g* for 15 min at 4°C. Proteins were separated in PAGE-SDS gels and blotted to PVDF membranes for detection with specific antibodies. Proteins of interest were detected by ECL (Pierce). We analyzed Western blots quantitatively using the NIH ImageJ program.

### Biotin-Switch Assay

Hundred microgram of total protein obtained from cellular lysates from U87-CM or IL-8 treated cells were denatured with sodium dodecyl sulfate (SDS) in the presence of methyl methanothiosulfonate (MMTS) (Jaffrey and Snyder, 2001). After acetone precipitation to remove excess MMTS, 1 mM ascorbate and biotin-HPDP (*N*-[6-(biotinamido) hexyl]-3′-(2′-pyridyldithio) propionamide) were added to reduce the S–NO bond and label the reduced thiol with biotin, respectively. Biotinylated proteins were captured with streptavidin–agarose beads and then separated by SDS-PAGE and detected with specific antibodies. Proteins of interest were detected by Western blotting.

### Endothelial Permeability Assay

Cells were grown on fibronectin-coated polycarbonate membranes (12-mm diameter, 0.4-μm pore size; Costar), for 5 to 6 days to achieve confluence. The day of the experiment, membranes were placed in a Navicyte system (San Diego, CA, United States). Luminal and abluminal chambers (Snapwell^TM^ Diffusion Chamber 66-0008; 5 ml each side) were filled with DMEM without phenol red. After a 15 min equilibration period, the luminal chamber was loaded with FITC-Dx-70 (final concentration 2.4 mg/ml). Samples for baseline permeability (20 μl) were obtained every 5 min for a period of 30 min. After addition of IL-8 or U87-CM, samples (20 μl) were obtained for an additional 30-min period. In the experiments using L-NMA (300 μM), the NOS inhibitor was added for 1 h while the cells were in the Navicyte system before agonist stimulation. In the experiments using ODQ (10 μM), the inhibitor was added 10 min before agonist stimulation. In the experiments using MAB208, IL-8 100 nM was incubated with MAB208 30 ng/ml for 5 min at 37°C before adding to the Navicyte system. In the case of U87-CM, 50 μl of U87-CM were incubated with 17 ng/ml MAB208 for 5 min at 37°C before adding to the Navicyte system. FITC-Dx-70 was measured using a spectrofluorometer. The permeability to FITC-Dx-70 was determined according to the Fick equation (Bekker et al., 1989; Hatakeyama et al., 2006; Marin et al., 2012).

### Immunofluorescence Microscopy

We followed established protocols for the detection of VE-cadherin (Marin et al., 2012; Guequen et al., 2016). After incubation with the corresponding primary and secondary antibodies, images were obtained using an epifluorescence microscope (Axioscop; Carl Zeiss) equipped with a 100× oil immersion objective lens and Axio Vision Rel. software (Zeiss, Germany). Eight-bit images were prepared for publication in Adobe Photoshop. Regions of interest were drawn around the plasma membrane and quantified using the ImageJ Program.

### *In vivo* Studies

Protocols for experiments on animals were approved by the Institutional Bioethics and Biosecurity Committee of the Universidad Austral de Chile and conducted according to NIH Guidelines for the use of animals in research. We measured microvascular permeability to macromolecules by intravital microscopy and S-nitrosylation of VE-cadherin and p120 in mouse cremaster muscle according to our published methods (Bekker et al., 1989; Marin et al., 2012; Guequen et al., 2016). Briefly, Rockefeller male mice (30–50 g) were anesthetized and the left cremaster muscle prepared for intravital microscopy by exposing the tissue and placing it in an *ad hoc* observation chamber, under constant superfusion with bicarbonate buffer. FITC-Dextran 70 kDa (100 mg/kg i.v.) was injected as fluorescent macromolecular tracer. Plasma to tissue transport was evaluated *in situ* by integrated optical intensity. Conditioned medium from U87 cells was added i.v. in the jugular vein and the extravasation of macromolecules was recorded for 15 min. For S-nitrosylation experiments, 1 μM IL-8 was added i.v, for 3 min and the left cremaster muscle was quickly dissected and homogenized in lysis buffer to measure protein S-nitrosylation. The animals were kept warm and anesthetized with supplementary doses of Nembutal as required. At the end of the experiment, mice were killed by an anesthetic overdose (∼300 mg/kg iv), followed by pneumothorax.

### Statistical Analysis

Experiments were conducted in groups with minimum *n* = 5. Data are expressed as mean ± standard error. Apparent differences were assessed for statistical significance using GraphPad Prism and Sigma Plot software. Significance was accepted at *p* < 0.05. Details of the specific statistical methods are indicated in each figure.

## Results

### Glioblastoma Tumors Show High Levels of S-Nitrosylation

**FIGURE 1:**
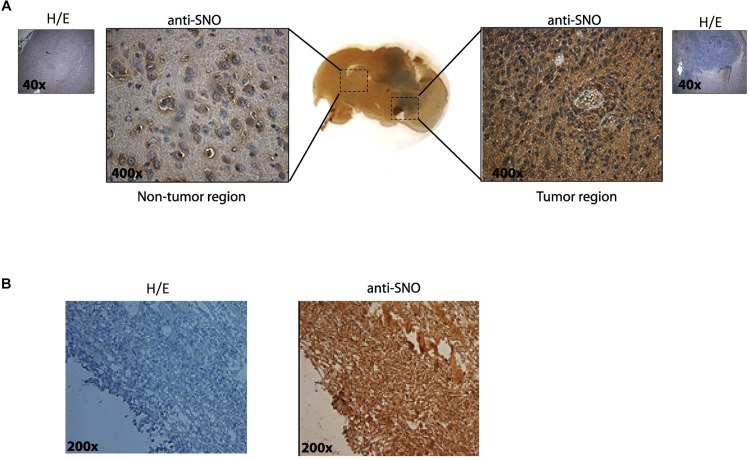
Increased S-nitrosylation is present in tumors in two *in vivo* models. **(A)** Glioma brain tumors were generated in Sprague Dawley rats by intrathecal inoculation of rat C6 glioma cells. Slices including non-tumor region and tumor regions were stained with H/E (purple/pink colors) and anti- S-nitrosylation (SNO) antibody (brown color). A representative immunohistochemistry is shown in the insets (without-primary antibody). Original magnification 40× and 400×. **(B)** S-nitrosylation in Glioblastoma subcutaneous tumors generated by inoculation of human U87MG-GSCs in NOD/SCID-IL2Rγ^*null*^ mice. A representative immunohistochemistry is shown in the left panel (without-primary antibody) and staining with antibody raised against SNO-cysteine in the right panel. Original magnification 200×.

We first determined qualitatively whether or not S-nitrosylation was present in GBM tumors. As a first approach, syngeneic tumors were induced in rats by intrathecal inoculating of C6 rat glioma cells. Rats were euthanized 14 days later, and brains were removed for histopathological analysis using anti-SNO-cysteine antibody (that reacts with all the S-nitrosylated proteins). Hematoxylin-eosin (H/E) staining without primary antibody shows the tumoral region (Figure 1A, insets). A basal level of SNO-cysteine staining was observed in non-tumor regions. Importantly, anti SNO-cysteine staining was remarkably dominant in tumor regions compared to non-tumor regions (Figure 1A). H/E was also more prevalent in the tumor region in Figure 1A. Subsequently, we inoculated subcutaneously human U87MG derived glioblastoma stem like-cells (GSCs) in NOD/SCID-IL2Rγ^*null*^ mice (Torres et al., 2016). Mice were euthanized 17 days later, and tumors were removed for histopathological analysis. Figure 1B shows H/E staining without primary antibody (left panel) and a high SNO staining in the same region (right panel). These results demonstrate that high levels of S-nitrosylation of proteins are present in the tumoral microenvironment of GBM.

### Conditioned Medium Obtained From the Glioblastoma Cell Line U87 (U87-CM) Culture Increases Endothelial Permeability Through eNOS Signaling and Suggest a Mechanism Dependent on the S-Nitrosylation Pathway

**FIGURE 2:**
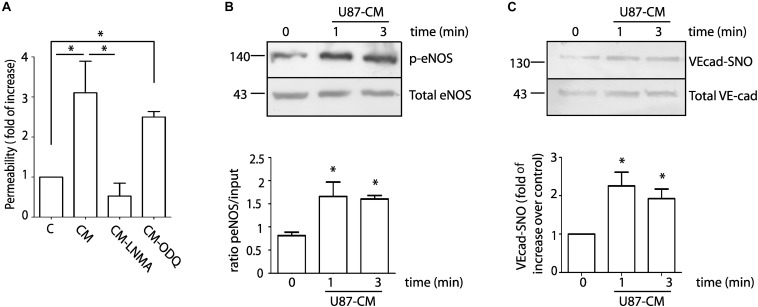
U87-CM increases permeability through endothelial nitric oxide synthase signaling involving S-nitrosylation of VE-cadherin and p120. **(A)** U87-CM (CM) increases permeability to FITC-dextran-70 across confluent EAhy926 monolayers relative to endothelial cells treated with control medium (C, DMEM 2% FCS). Inhibition of eNOS with L-NMA blocked U87-CM-induced hyperpermeability, but inhibition of sGC-PKG with ODQ did not block hyperpermeability. One-way ANOVA and Newman–Keuls test. ^*^p < 0.05; *n* = 5. **(B)** U87-CM phosphorylates eNOS at Ser 1177. EAhy926 cells were incubated with U87-CM for 1 and 3 min. Protein extracts were processed for western blot to detect phosphorylation. One-way ANOVA and Tukey’s Multiple Comparison Test. ^*^p < 0.05 compared with time 0 min; *n* = 5. **(C)** U87-CM S-nitrosylates VE-cadherin. EAhy926 cells were incubated with U87-CM for 1 and 3 min and processed for biotin switch assay to detect S-nitrosylation. One-way ANOVA and Tukey’s Multiple Comparison Test ^*^p < 0.05 compared with time 0 min; *n* = 5.

To investigate in detail the role of NO in endothelial cell function, we treated EAhy926 (immortalized human umbilical vein endothelial cells) with U87-CM and measured permeability to FITC-dextran 70 (molecular weight: 70 kDa) by dextran extravasation assay. Figure 2A shows that U87-CM increases endothelial permeability 3.11 ± 0.79 times over control. To test whether endothelial hyperpermeability depends on eNOS activation, we treated cells with 300 μM NG-methyl-L-arginine (L-NMA), a global nitric oxide synthase inhibitor. Pre-treatment with L-NMA for 1 h completely blocked U87-CM-induced hyperpermeability. Since NO can independently activate the soluble guanylate cyclase-protein kinase G (sGC-PKG) pathway and the S-nitrosylation pathway, we applied ODQ (1H-[1,2,4]oxadiazolo[4,3,-a]quinoxalin-1-one), a recognized sGC-PKG inhibitor, to differentiate between these two pathways. Inhibition of sGC-PKG did not block endothelial hyperpermeability induced by U87-CM, suggesting that this process depends on S-nitrosylation (Moldogazieva et al., 2018). To corroborate the involvement of eNOS in this process, we measured eNOS phosphorylation at Ser 1177 as an index of enzyme activation. As eNOS activation occurs within minutes during hyperpermeability (Sanchez et al., 2008, 2009), we analyzed its phosphorylation at Ser 1177 at 1 and 3 min post stimulation of EAhy926 cells with U87-CM. As it is shown in Figure 2B, eNOS was activated at both times. It has been demonstrated that VE-cadherin is internalized in response to U87-CM (Dwyer et al., 2012) and we previously demonstrated that VE-cadherin becomes S-nitrosylated in correlation with its internalization and induction of endothelial permeability (Guequen et al., 2016). Therefore, we investigated whether U87-CM treatment induces VE-cadherin S-nitrosylation. Stimulation for 1 and 3 min induced VE-cadherin S-nitrosylation (Figure 2C). All these results indicated that U87-CM-induced hyperpermeability involves eNOS activation leading to VE-cadherin S-nitrosylation as previously reported for other stimuli (Guequen et al., 2016).

### Blockade of IL-8 Inhibits Vascular Hyperpermeability and S-Nitrosylation of VE-Cadherin Induced by U87-CM

**FIGURE 3:**
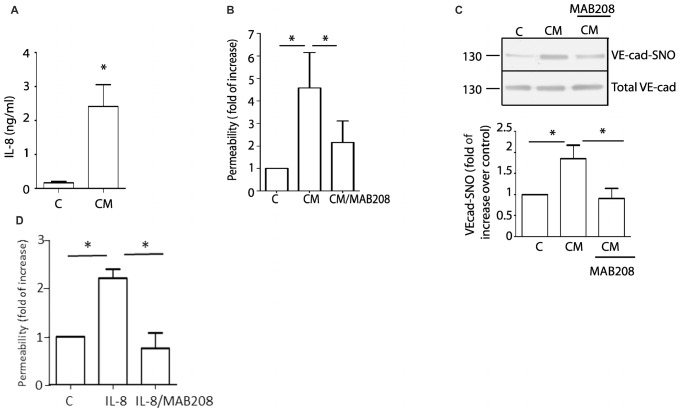
Blocking IL-8 inhibits U87-CM induced endothelial hyperpermeability and S-nitrosylation of VE-cadherin. **(A)** ELISA measurements of IL-8 in U87-CM. **(B)** U87-CM increases endothelial permeability mainly due to the presence of IL-8, as this effect is abrogated by blocking IL-8 with MAB208. One-way ANOVA and Bonferroni’s Multiple Comparison Test.^*^ *p* < 0.05; *n* = 7. **(C)** U87-CM induces S-nitrosylation of VE-cadherin through IL-8. Blocking IL-8 with MAB208 abolishes CM-induced S-nitrosylation of VE-cadherin. One- way ANOVA and Bonferroni’s Multiple Comparison Test, ^*^p < 0.05; *n* = 5. **(D)** MAB208 specifically blocks the effect of IL-8 in endothelial permeability. One-way ANOVA and Tukey’s Multiple Comparison Test.^*^p < 0.05; *n* = 5.

Glioblastoma patients have high levels of circulating IL-8 (Albulescu et al., 2013). In addition, U87-CM induced hyperpermeability has been associated with the presence of IL-8 in U87-secretome (Dwyer et al., 2012). We confirmed this observation measuring IL-8 levels by ELISA. The levels of IL-8 in conditioned medium was 2.41 ± 0.64 ng/ml (Figure 3A). We additionally confirm the presence of IL-8 in conditioned medium using MAB208, a specific IL-8 blocking antibody (Masood et al., 2001), which significantly inhibited U87-CM induced hyperpermeability (Figure 3B). Concordant with the causal relationship between VE-cadherin S-nitrosylation and endothelial barrier disruption, MAB208 also caused a statistically significant inhibition of U87-CM induced VE-cadherin S-nitrosylation (Figure 3C). To test the specific effect of MAB 208 on IL-8 we measured endothelial permeability in response to IL-8 in the presence of MAB208. Figure 3D shows that MAB208 specifically blocked the effect of IL-8. All together, these results suggest that IL-8 secreted by U87 GBM cells induces hyperpermeability and S-nitrosylation of VE-cadherin.

### IL-8 Induces Endothelial Hyperpermeability Through NO Signaling and Induces S-Nitrosylation of VE-Cadherin and p120

**FIGURE 4:**
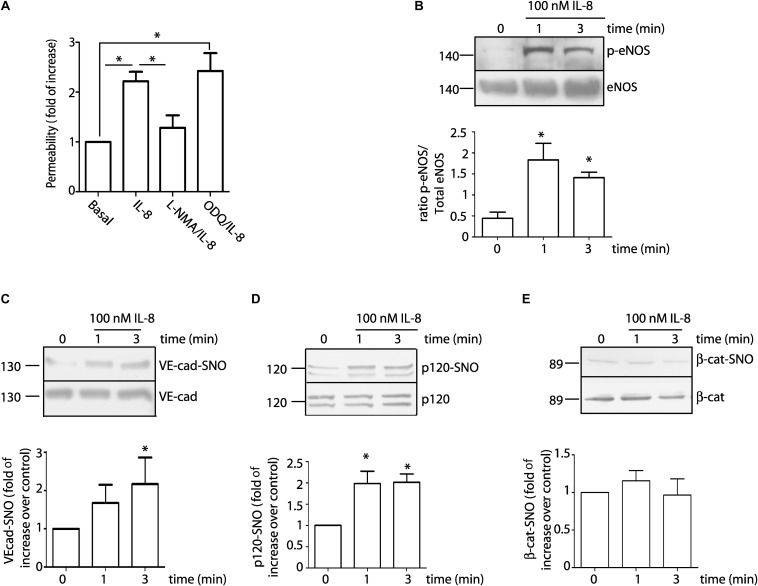
100 nM IL-8 increases endothelial permeability through eNOS signaling involving S-nitrosylation of VE-cad and p120. **(A)** IL-8 increases permeability to FITC-dextran-70 across confluent EAhy926 monolayers. Inhibition of eNOS with L-NMA, but not inhibition of sGC with ODQ, inhibits IL-8 induced hyperpermeability. One-way ANOVA and Bonferroni’s Multiple Comparison Test, ^*^P < 0.05; *n* = 5. **(B)** IL-8 significantly increases phosphorylation of eNOS at Ser 1177 as a function of time. One-way ANOVA and Newman–Keuls test. ^*^P < 0.05; *n* = 5. IL-8 significantly increased S-nitrosylation of VE-cadherin **(C)** and p120 **(D)**, however, IL-8 did not cause S-nitrosylation of β-catenin **(E)**. ^*^P < 0.05 compared with time 0 min; *n* = 5; One-way ANOVA and Bonferroni’s Multiple Comparison Test.

Since IL-8 blocking antibody inhibits endothelial hyperpermeability and S-nitrosylation of VE-cadherin, we examine whether recombinant IL-8 would reproduce the effects of the U87-CM. EAhy926 cells were treated with 100 nM IL-8. Figure 4A shows that IL-8 significantly increased endothelial permeability (2.2 ± 0.2 fold relative to control). Pre-treatment of the cells with L-NMA blocked IL-8 induced hyperpermeability, while inhibition of sGC-PKG with ODQ failed to block IL-8 induced hyperpermeability. Similar to the findings with U87-CM, IL-8 also increased eNOS phosphorylation in EAhy926 cells as soon as 1 min after its application, and the enzyme remained phosphorylated for at least 3 min (Figure 4B). These results support the concept that IL-8 causes hyperpermeability through NO dependent signaling. In this regard, we and others previously showed that, p120, β-catenin and VE-cadherin are S-nitrosylated during the process of induction of endothelial hyperpermeability, in response to proinflammatory agents: VEGF, PAF, TNF-α (Thibeault et al., 2011; Marin et al., 2012; Guequen et al., 2016). The S-nitrosylation of these proteins correlates with the breakdown of the adherens junction complex and internalization of these proteins (Marin et al., 2012; Guequen et al., 2016). Figures 4C,D show that IL-8, applied for 1 and 3 min, induces S-nitrosylation of VE-cad and p120. At difference with previous reports, IL-8 it did not S-nitrosylated β-catenin in EAhy926 cells (Figure 4E).

### IL-8 Induces NO-Dependent VE-Cadherin Internalization and Disruption of the Adherens Junction Complex

**FIGURE 5:**
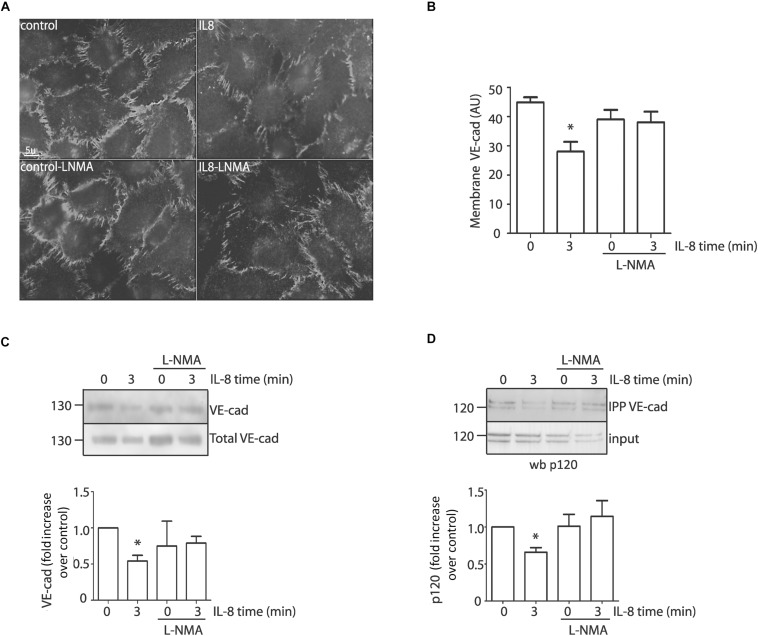
IL-8 induces VE-cadherin internalization and disruption of the adherens junction complex through eNOS signaling in EAhy926 monolayers. **(A)** Internalization of VE-cadherin induced by IL-8 was evidenced by indirect immunofluorescence in confluent cells. Inhibition of eNOS with L-NMA blocks IL-8 induced VE-cadherin internalization and retains the fluorescent label at the cell membrane. Scale bar: 5 μm. **(B)** Quantification of VE-cadherin at the plasma membrane (AU = arbitrary units) by image analysis. ^*^P < 0.05, Two -way ANOVA and Holm-Sidak as *post hoc* test, *n* = 5. **(C)** VE-cadherin location examined by cell surface biotinylation. IL-8, applied for 3 min, significantly reduced VE-cadherin at the cell surface. Pretreatment of the monolayers with L-NMA inhibited IL-8 induced VE-cadherin internalization. ^*^P < 0.05, Two -way ANOVA and Holm-Sidak as *post hoc* test, *n* = 5. **(D)** Association between VE-cadherin and p120 by co-immunoprecipitation. EAhy926 cells stimulated with IL-8 were immunoprecipitated with VE-cadherin antibody and probed for p120. IL-8 disrupts the binding between VE-cadherin and p120. IL-8 induced disruption was inhibited by pretreating the monolayers with L-NMA. ^*^P < 0.05 compared with control; *n* = 5. Two-way ANOVA and Duncan’s method as a post test.

Because IL-8 induces VE-cadherin internalization in association with an increase in permeability (Gavard et al., 2009), our next step was to test whether NO regulates VE-cadherin internalization in EAhy926 cells. We analyzed changes in localization of VE-cadherin in response to IL-8 in the presence or absence of L-NMA. Figure 5A shows the localization of VE-cadherin in control EAhy926 cells and after IL-8 treatment. In control confluent cells VE-cadherin is distributed predominantly along plasma membrane. IL-8 causes decreased fluorescence intensity in the plasma membrane at 3 min, while the inhibition of eNOS with L-NMA significantly reduced this effect. Figure 5B shows the image analysis quantification of immunofluorescence at the plasma membrane. To further test the regulation of membrane VE-cadherin by IL-8, we performed studies using cell surface biotinylation. Figure 5C shows cell surface biotinylation in control and after IL-8 treatment. Western blotting indicates an IL-8 induced decrease in VE-cadherin at the plasma membrane, which was inhibited by pre-treatment of the cells with L-NMA. VE-cadherin remains in the plasma membrane mainly by binding to p120 that blocks an endocytosis signal in VE-cadherin (Nanes et al., 2012); thus, in order to be internalized VE-cadherin has to be separated from p120. We used co-immunoprecipitation as an approach to assess the influence of IL-8 on protein-protein interactions at the adherens junctions. IL-8 applied for 3 min reduced the amount of p120 that co-immunoprecipitated with VE-cadherin indicating disruption of the protein-protein interaction (Figure 5D). The disruptive effect of IL-8 was inhibited by L-NMA suggesting that this action is associated with eNOS-derived NO (Figure 5D).

### Effects of IL-8 and U87-CM on Endothelial Hyperpermeability *in vivo*

**FIGURE 6:**
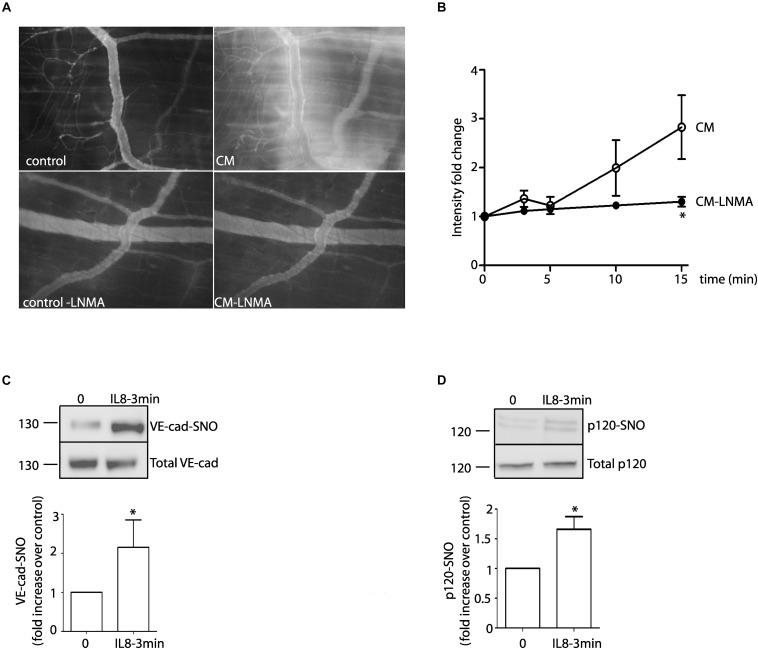
*In vivo* effects of U87-CM and IL-8. **(A)** U87-CM increased permeability to FITC-dextran 70 in the mouse cremaster muscle. Pre-treatment with L-NMA blocked U87-CM-induced hyperpermeability (bottom panel). **(B)** Time course of FITC-Dextran 70 leakage in the mouse cremaster, as determined by intensity fold change in the interstitium. ^*^p < 0.05 compared to U87-CM; *n* = 5. Two-way ANOVA and Bonferroni’s Multiple Comparison Test. Recombinant IL-8 (1 μM) i.v applied for 3 min induces S-nitrosylation of VE-cadherin **(C)** and p120 **(D)** in mouse cremaster muscle. (Un-paired Student’s *t*-test, *n* = 5, ^*^p < 0.05).

Previous studies have used the cremaster muscle of rat and mouse as models to investigate increased permeability in response to human malignant ascites (Heuser et al., 1988; White et al., 1988). Even though tumors normally develop in other vascular territories, the cremaster muscle can be used as a model to investigate changes in vascular permeability related to tumor biology. To test the significance of our *in vitro* results, we analyzed whether or not IL-8 and U87-CM induce S-nitrosylation and hyperpermeability, respectively, in the mouse cremaster muscle using intravital microscopy. Figure 6A shows that U87-CM induces microvascular hyperpermeability to FITC-dextran 70. Inhibition of NOS activity with L-NMA blocked this effect. Figure 6B shows the time course of the changes in interstitial intensity as an index of the increase in microvascular permeability. IL-8, administered i.v., in the jugular vein induced S-nitrosylation of VE-cadherin and p120 in the mouse cremaster (Figures 6C,D). These results indicate that *in vivo* U87-CM increases microvascular permeability and IL-8 increases S-nitrosylation of junctional proteins.

## Discussion

The disruption of the endothelial barrier causes a marked increase in endothelial permeability, a key event in the pathophysiology of many inflammatory diseases. Increased vascular permeability contributes importantly to the progression of GBM, an aggressive and deadly brain tumor (Jain et al., 2007; Sorensen et al., 2009; Rahman et al., 2010; Lim et al., 2011; Torres et al., 2017), however, the specific mechanisms are not completely understood. We report that intrathecal rat glioma and subcutaneous human GBM tumors revealed strong S-nitrosylation in tumoral tissue. U87-CM activated the eNOS-NO derived pathway and induced S-nitrosylation of VE-cadherin and endothelial hyperpermeability to macromolecules. Both effects, S-nitrosylation and permeability, were blocked in the presence of blocking antibody for IL-8. Recombinant IL-8 induced endothelial hyperpermeability through NO signaling in association with S-nitrosylation of VE-cadherin and p120 but not of β-catenin. IL-8 induced VE-cadherin internalization and disruption of the interaction between VE-cadherin and p120, an effect that was blocked in the presence of NOS inhibitor. *In vivo*, IL-8 induced S-nitrosylation of VE-cadherin and p120 in the mouse cremaster. Also, U87-CM increased endothelial permeability and this effect was blocked by the NOS inhibitor L-NMA.

The regulation of cellular process by NO and S-nitrosylation strictly depends on the localization of NOS isoforms (Iwakiri et al., 2006; Marin et al., 2012; Zamorano et al., 2017). In basal conditions (non-stimulation) eNOS localizes preferentially to caveolae and interacts with caveolin-1, which keeps eNOS activity at basal levels (Feron and Balligand, 2006). Upon agonist stimulation, eNOS is internalized and located to the cytosol to promote endothelial permeability in venules (Sánchez et al., 2006; Sanchez et al., 2008, 2009). This different localization of eNOS in basal and stimulated conditions might explain the fact that different studies have shown that inhibition of constitutive NO increases microvascular permeability (Kubes and Granger, 1992; Kurose et al., 1993; Predescu et al., 2005). The regulation of endothelial permeability by eNOS in basal conditions with low level of NO production at the caveolae could be totally different than in stimulated conditions, where eNOS moves to the cytosol and also releases higher concentrations of NO. In fact, the effects of basal NO in permeability seems to be mediated by sGC-PKG pathway (Kurose et al., 1993), whereas the effects of NO induced by pro-inflammatory stimulus strongly point to S-nitrosylation as the regulatory signaling mechanism (Thibeault et al., 2011; Marin et al., 2012, Guequen et al., 2016; Zamorano et al., 2017, 2019).

Our results support the dependence of VE-cadherin internalization and associated hyperpermeability on U87-CM and IL-8 (Dwyer et al., 2012). We advance our knowledge of the field by demonstrating that U87-CM and IL-8, its main content, cause junctional protein S-nitrosylation in association with endothelial hyperpermeability. The role of S-nitrosylation in cancer has been studied mainly in tumoral cells. In particular, proteomic approaches have led to the identification of the S-nitrosoproteome in lung carcinoma cells (Ben-Lulu et al., 2017); prostate carcinoma cell lines (Lam et al., 2010) and in patients with colorectal carcinoma (Chen et al., 2014). S-nitrosylation of targets such as src, EGF-R, ras and other proteins activates different pathways leading to metastasis (Rahman et al., 2009; Switzer et al., 2012). In GBM, microglial caspase-3 activity is inhibited by iNOS-induced S-nitrosylation causing a pro-tumoral microenvironment (Shen et al., 2016). Similarly, PTEN activity is inhibited by iNOS-induced S-nitrosylation leading to reduced suppressor tumor activity (Yu et al., 2005), an important observation since PTEN mutations are markers for GBM (Li et al., 1997; Haas-Kogan et al., 1998). In spite of these advances, the function of S-nitrosylation in endothelial barrier function in cancer and its contribution to tumoral progression has not been investigated. Histological analysis of patients show a marked increase in iNOS and nNOS immunoreactivity in tumor cells of glial neoplasms correlating with degree of malignancy (Garbossa et al., 2001; Broholm et al., 2003). Our histological analysis of glioma and GBM tumors *in vivo* revealed high levels of S-nitrosylation in tumoral tissue (Garbossa et al., 2001; Broholm et al., 2003) corroborating that NOS activity is elevated in GBM. Our study is the first one to demonstrate that the proinflammatory factor IL-8 secreted by GBM tumor cells induces S-nitrosylation of key junctional proteins that regulate vascular permeability in endothelial cells. Our results are due to activation of eNOS and eNOS-dependent NO production. This finding agrees with the work from Broholm et al. (2003) showing increased eNOS expression in endothelial cells of the tumor vasculature in glioma tumors (Broholm et al., 2003). In addition, we reported that iNOS and nNOS do not substitute for eNOS in triggering the onset of hyperpermeability in response to pro-inflammatory agonists (Hatakeyama et al., 2006; Sanchez et al., 2009).

High endothelial permeability contributes to interstitial deposition of plasma proteins, which may provide a provisional matrix for the inward migration of fibroblasts and endothelial cells into tumors favoring angiogenesis and tumor growth. Furthermore, tumoral cells can access the leaky microvasculature and disseminate to other organs contributing to metastases (Dvorak et al., 1988). Because several inflammatory mediators share NO as a common signaling pathway, strategies based on regulating NOS-dependent S-nitrosylation could be more effective than blocking each cytokine independently. Targeting inhibition of S-nitrosylation offers also the advantage to avoid collateral effects such as hypertension, which can be induced by general NOS inhibitors. It is tempting to advance the concept that blocking S-nitrosylation may be a target to fight GBM, an aggressive cancer. While NOS inhibition in rats bearing C6 glioma decreased tumor volume (Swaroop et al., 2000), specific blockade of S-nitrosylation seems to be a better alternative than blocking any and all NOS, inasmuch as genetic deletion of eNOS delayed but did not prevent development of cancer (Gratton et al., 2003).

## Data Availability

The raw data supporting the conclusions of this manuscript will be made available by the authors, without undue reservation, to any qualified researcher.

## Ethics Statement

This study was carried out in accordance with the recommendations of Institutional Animal Care and Use Committee at the Universidad Austral de Chile according to the NIH Guide for the Care and Use of Laboratory Animals. The protocol was approved by the Institutional Animal Care and Use Committee at the Universidad Austral de Chile according to the NIH Guide for the Care and Use of Laboratory Animals.

## Author Contributions

AG, PZ, FC, TK, and AT contributed with experiments and data analysis. PE contributed with experiments, data analysis, discussion, and writing. MB, FS-O, JG, WD, and CQ contributed with discussion and writing. JS and FS contributed with experiments design, experiments, data analysis, discussion, and writing.

## Conflict of Interest Statement

The authors declare that the research was conducted in the absence of any commercial or financial relationships that could be construed as a potential conflict of interest.

## Abbreviations

EAhy926: immortalized human umbilical vein endothelial cells
eNOS: endothelial nitric oxide synthase
GBM: glioblastoma
H/E: hematoxylin-eosin
IL-8: interleukin-8
L-NMA: NG-methyl-L-arginine
NO: nitric oxide
SNO: S-nitrosylation
U87-CM: conditioned medium from U87 cells
VE-cad: VE-cadherin
